# Application of a Low-Cost mHealth Solution for the Remote Monitoring of Patients With Epilepsy: Algorithm Development and Validation

**DOI:** 10.2196/50660

**Published:** 2023-12-19

**Authors:** Natarajan Sriraam, S Raghu, Erik D Gommer, Danny M W Hilkman, Yasin Temel, Shyam Vasudeva Rao, Alangar Satyaranjandas Hegde, Pieter L Kubben

**Affiliations:** 1 Center for Medical Electronics and Computing Ramaiah Institute of Technology Bengaluru India; 2 Department of Neurosurgery Maastricht University Maastricht Netherlands; 3 Department of Clinical Neurophysiology Maastricht University Medical Centre Maastricht Netherlands; 4 Institute of Neuroscience Ramaiah Medical College and Hospitals Bengaluru India

**Keywords:** Android, epileptic seizures, mobile health, mHealth, mobile phone–based epilepsy monitoring, support vector machine, seizure, epileptic, epilepsy, monitoring, smartphone, smartphones, mobile phone, neurology, neuroscience, electroencephalography, EEG, brain, classification, detect, detection, neurological, electroencephalogram, diagnose, diagnosis, diagnostic, imaging

## Abstract

**Background:**

Implementing automated seizure detection in long-term electroencephalography (EEG) analysis enables the remote monitoring of patients with epilepsy, thereby improving their quality of life.

**Objective:**

The objective of this study was to explore an mHealth (mobile health) solution by investigating the feasibility of smartphones for processing large EEG recordings for the remote monitoring of patients with epilepsy.

**Methods:**

We developed a mobile app to automatically analyze and classify epileptic seizures using EEG. We used the cross-database model developed in our previous study, incorporating successive decomposition index and matrix determinant as features, adaptive median feature baseline correction for overcoming interdatabase feature variation, and postprocessing-based support vector machine for classification using 5 different EEG databases. The Sezect (Seizure Detect) Android app was built using the Chaquopy software development kit, which uses the Python language in Android Studio. Various durations of EEG signals were tested on different smartphones to check the feasibility of the Sezect app.

**Results:**

We observed a sensitivity of 93.5%, a specificity of 97.5%, and a false detection rate of 1.5 per hour for EEG recordings using the Sezect app. The various mobile phones did not differ substantially in processing time, which indicates a range of phone models can be used for implementation. The computational time required to process real-time EEG data via smartphones and the classification results suggests that our mHealth app could be a valuable asset for monitoring patients with epilepsy.

**Conclusions:**

Smartphones have multipurpose use in health care, offering tools that can improve the quality of patients’ lives.

## Introduction

According to the International League Against Epilepsy, epileptic seizures are characterized by an unpredictable occurrence pattern and transient dysfunctions of the central nervous system due to excessive and synchronous abnormal neuronal activity in the cortex [1]. Electroencephalography (EEG) can be used to determine the epileptogenic zone or to monitor patients in the intensive care unit for seizures or monitor seizures for therapy adjustment. EEG signals are collected over a period of time and analyzed to detect seizure events. Today, almost everyone uses smartphones, and smartphone apps are being used to solve real-world human challenges including health-related issues. Regarding the remote monitoring of patients with epilepsy, there is a need to develop an efficient smartphone app that processes long-term EEG recordings for seizure detection. Therefore, the goal of this paper was to develop and evaluate the feasibility of a mobile app for the remote monitoring of patients with epilepsy.

In this context, an automatic mobile phone–based approach for epileptic seizure detection was proposed by Menshawy et al [2] using time, frequency, entropy, and discrete wavelet transform–based features with k-means clustering. EEG signals recorded from the EEG headset were stored in smartphones and transmitted to a server. The preprocessing, feature extraction, feature normalization, feature selection, and classification model of EEG signals were performed on a cloud server. The results were sent to the smartphones of patients and physicians via a backend server. Based on the classification results, caretakers were notified to take appropriate action. This study faced limitations in terms of memory as the complete EEG signal had to be sent to the server. Additionally, this approach was computationally expensive due to the use of a large number of features. McKenzie et al [3] assessed the ability of Smartphone Brain Scanner-2 to detect epileptiform abnormalities using an Android tablet that was wirelessly connected to a 14-electrode EasyCap headset. An Android-based smartphone app for monitoring patients with epilepsy was proposed using subband features and a support vector machine (SVM) classifier [4]. mHealth (mobile health) has been proposed to detect generalized tonic-clonic seizures, whereby an alarm is triggered for timely interventions resulting in a possibly reduced risk of sudden unexpected death in epilepsy [5].

Kiral-Kornek et al [6] proposed a mobile system–based epileptic seizure prediction using big data and deep learning using intracranial EEG signals. Typical statistics like seizures per month, average sensitivity, and average warning time were reported. Moreover, other studies have proposed a cloud-based alert system using advanced statistics [7] and have explored seizure prediction through deep learning techniques for EEG big data [8,9]. Some studies [2,6-14] have used cloud computing for EEG analysis and seizure detection. Additionally, a few mobile devices, namely SmartWatch, Embrace Watch, Brain Sentinel, and EpiWatch App, have been developed for seizure detection to alert caretakers and to prevent sudden unexpected death due to epilepsy [15].

**Figure 1:**
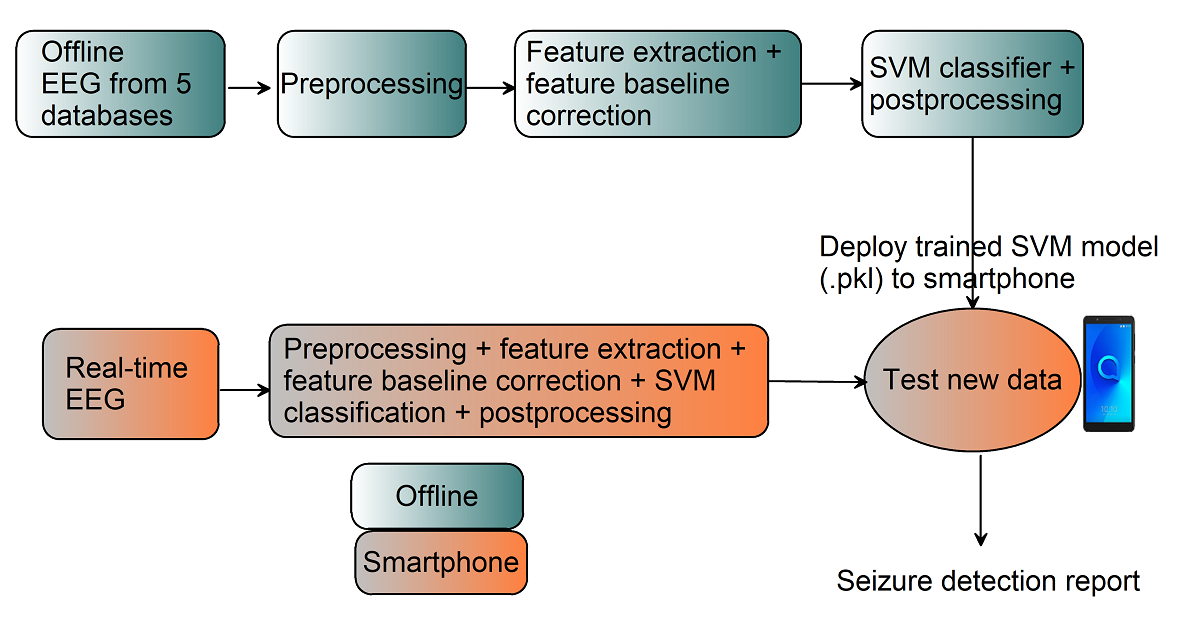
Block diagram of the proposed smartphone-based monitoring approach for patients with epilepsy. EEG: electroencephalography; SVM: support vector machine.

Our study focuses on harnessing smartphone capabilities to implement the entire seizure detection model, which eliminates the need for cloud technology. The Sezect (Seizure Detect) app, our mobile phone–based seizure detection model, provides information such as the number of channels, sampling frequency, EEG signal duration, seizure frequency per channel, and seizure-affected channels. Further, the app was developed using open-source software, allowing researchers public access and the ability to replicate the process. Therefore, the proposed approach could be a valuable tool for the remote monitoring of patients with epilepsy. Figure 1 shows a block diagram of the proposed smartphone-based monitoring approach for patients with epilepsy.

## Methods

### Clinical EEG Recordings

In order to deploy the seizure detection model via smartphone, the cross-database model in our previous study was developed using EEG recordings from Ramaiah Medical College and Hospitals (RMCH) (Bengaluru, India), Children’s Hospital Boston-Massachusetts Institute of Technology (CHB-MIT) (Boston, MA), Temple University Hospitals (TUH) (Philadelphia, PA), Maastricht University Medical Centre (MUMC) (Maastricht, Netherlands), and the University of Bonn (UBonn) (Bonn, Germany) [16]. The same cross-database model was implemented on a smartphone and validated using 20 new patients’ EEG recordings collected from the RMCH and MUMC databases. EEG recordings with a total duration of 13 hours were tested via smartphone.

### Ethical Considerations

The 3 EEG recordings used in our study, namely from CHB-MIT, TUH, and UBonn, are available publicly. Ethical committee approval was sought for the RMCH and MUMC EEG recordings before use in this study.

### Chaquopy

Chaquopy is the Python software development kit for Android [17], which allows reuse of existing Python code on Android and takes advantage of Python Package Index packages including *numpy*, *sci-kit learn*, *scipy*, and others. The GitHub repository provides more details on how to use Chaquopy [18]. The *chaquopy-console* template was used to run the seizure detection Python code on the app.

### Seizure Detection Model

The methods followed in this study were introduced by us in our previous studies [16,19-21]. The optimized cross-database seizure detection model was built in our previous study [16]. Two features, the successive decomposition index [19] and matrix determinant [20], were extracted from all 5 databases and their baseline was updated using adaptive median feature baseline correction [21]. The features were classified using the SVM classifier via the leave-one-database-out cross-validation method and a postprocessing technique was implemented by applying a 10-tap moving average filter to the classifier output to reduce false detections. This model was then coded in Python and exported into a pickle file for smartphones to test the new EEG recordings.

### Mobile Phone–Based Seizure Monitoring

To demonstrate proof of concept, the Sezect Android app was tested for epileptic seizure detection using EEG signals. It is important to investigate how different versions or models of smartphones perform in processing EEG signals, which will be useful to know to make the proposed method scalable. Therefore, we tested the proposed algorithm on the following mobile phones: Nokia, Moto X Play, and Redmi Note 4. Overall, 20 new EEG recordings from patients with epilepsy from both MUMC and RMCH were used to evaluate the efficiency of these smartphones for seizure detection. Using *joblib* from the *sklearn* *externals* library, the trained SVM model was dumped into a .pkl file and loaded into the Sezect app to test the recordings.

## Results

**Figure 2:**
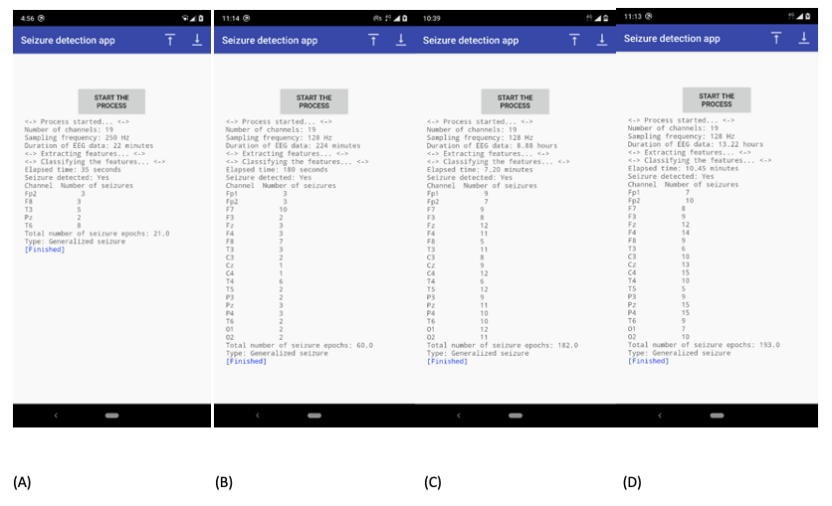
Screenshots of the Sezect app results: (A) the Maastricht University Medical Centre database with 22 minutes of electroencephalography (EEG) data and the Ramaiah Medical College and Hospitals database with (B) 3.73 hours, (C) 8.88 hours, and (D) 13.22 hours of EEG data.

The Sezect app was tested on 3 Android mobile phones with the following configurations: (1) Nokia 8.1 (Android 10), (2) Moto X Play (Android 8), and (3) Redmi Note 4 (Android 10). Screenshots of the Sezect app results using Nokia are shown in Figure 2, and a video of running the app is available online [22]. As shown in Figure 2, the app pulls information such as the number of channels, sampling frequency, and duration of the complete EEG data file. Further, it displays the elapsed time required to process the complete EEG file, the number of seizure events detected per channel, and the total number of seizure epochs (each epoch length is 10 seconds).

**Figure 3:**
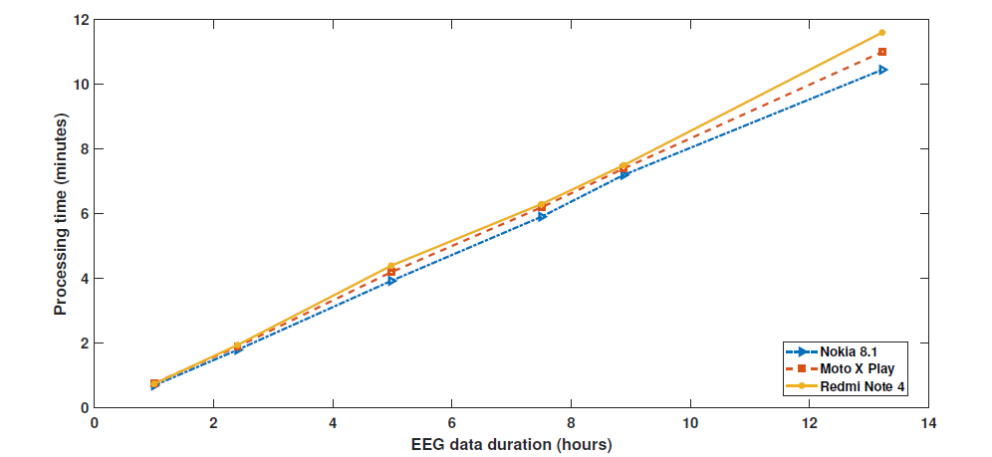
The processing time required to analyze and classify various durations of EEG signals on different smartphones. EEG: electroencephalography.

Figure 3 illustrates the time taken by various smartphones to process EEG recordings of different durations. The processing time of the Sezect app shows that a mobile platform is capable of handling large amounts of EEG data and perform feature calculation and classification. The various mobile phones did not differ substantially in processing time, which indicates a range of phone models can be used for implementation. Further, the robustness and scalability of the app was examined using various hardware configurations for all 3 smartphones. We observed a sensitivity of 93.5%, a specificity of 97.5%, and a false detection rate of 1.5 per hour for new EEG recordings using the Sezect app. The results suggest that our proposed seizure detection algorithm could be a valuable asset to remotely monitor patients with epilepsy using smartphone apps.

## Discussion

### Comparison With State-of-the-Art Studies

Few studies have used cloud technology, the internet of things, and smartphones to analyze EEGs and detect seizure epochs. Menshawy et al [2] used server-based processing for data preprocessing, feature engineering, and classification; subsequently, generated reports were sent to doctors, and caretakers were alerted upon detecting seizures. Cloud computing was effectively used in some studies [4,10,12,14] to perform feature engineering and classification using cloud technology. Moreover, mobile devices like SmartWatch, Embrace Watch, Brain Sentinel, and the EpiWatch App are in use, designed to detect specific types of seizures [15]. However, the proposed Sezect app was built using the cross-database model from 5 EEG databases and has been found to be effective in terms of computational time when tested on 3 different smartphones. Physicians and nurses working in rural areas can record EEG data and validate it using the Sezect app.

### Contributions

The following is a summary of our contributions:

1. We developed the Sezect app for Android using open-source software to remotely monitor patients with epilepsy. The app is made available as open-source software to improve the reproducibility of our results. The source code for the Sezect app can be found online [23].
2. The feasibility of smartphones for handling large EEG recordings was determined using the Sezect app. Further, we examined the time complexity by assessing the elapsed time of the mobile app across various EEG durations.
3. Running all tasks on the cloud demands substantial memory and can be costly. This study’s major contribution lies in demonstrating the feasibility of automated seizure detection via smartphones, eliminating the involvement of cloud infrastructure.

### Clinical Significance

Remote monitoring using smartphone apps will be useful to monitor patients with epilepsy by analyzing EEG signals collected over a period of time. Smartphones can serve multiple uses in health care to improve the quality of patients’ lives. The advanced technology of smartphones can be applied to solve the workload burden of experts.

### Future Directions

**Figure 4:**
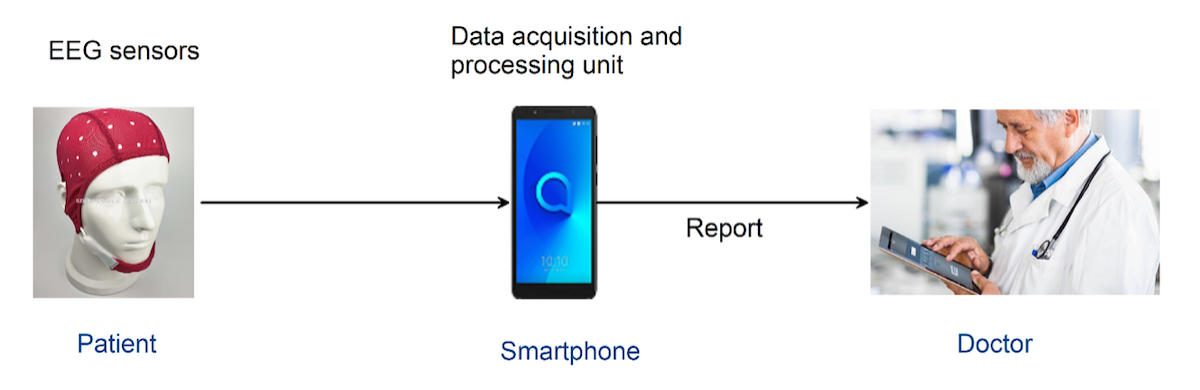
A future scope architecture for real-time smartphone-based seizure detection and the remote monitoring of patients with epilepsy. EEG: electroencephalography.

This study presented a proof of concept for a low-cost mHealth solution aimed at the automated detection of epileptic seizures for remote monitoring. Figure 4 illustrates the architectural scope for a future remote monitoring system. In such a system, a wireless EEG headset will be provided to the patient and continuous real-time EEG signals will be recorded and stored on smartphones. A cross-database classification model within the smartphone will analyze EEG signals, generating a report sent directly to the relevant physician for further action.

### Limitations

In our current implementation, we observed a slightly elevated false detection rate, which needs to be addressed in the future.

### Conclusion

The feasibility of a mobile phone–based app for the remote monitoring of patients with epilepsy using a database-independent optimized algorithm was demonstrated. The app is open source, allowing researchers to reproduce it according to their specific needs. It was tested using 3 different types of smartphones. The results suggest that smartphones are capable of handling large amounts of EEG data for feature calculation and classification.

## Abbreviations

CHB-MIT: Children’s Hospital Boston-Massachusetts Institute of Technology
EEG: electroencephalography
mHealth: mobile health
MUMC: Maastricht University Medical Centre
RMCH: Ramaiah Medical College and Hospitals
Sezect: Seizure Detect
SVM: support vector machine
TUH: Temple University Hospitals
UBonn: University of Bonn

## Data Availability

The EEG data used in this study are intended solely for research purposes and cannot be made available to the public.
